# Prevalence, under-reporting, and epidemiological surveillance of COVID-19 in the Araguaína City of Brazil

**DOI:** 10.1371/journal.pone.0300191

**Published:** 2024-06-05

**Authors:** Monike da Silva Oliveira, Rogério Fernandes Carvalho, Carolina Merlin Meurer, Ézio Machado Rodrigues, Bianca Pereira Dias, Isac Gabriel Cunha dos Santos, Cristiane Alves Nascimento, Yron Moreira Rodrigues, Alessandro José Ferreira dos Santos, Katyane de Sousa Almeida, Ueric José Borges de Souza, Fabrício Souza Campos, Juliane Ribeiro, Célia Maria de Almeida Soares, José Carlos Ribeiro Júnior

**Affiliations:** 1 Molecular Biology Laboratory, Institute of Biological Sciences, Federal University of Goiás, Goiânia, Goiás, Brazil; 2 Microbiology Laboratory, Federal University of North Tocantins, Araguaína, Tocantins, Brazil; 3 Bioinformatics and Biotechnology Laboratory, Federal University of Tocantins, Gurupi, Tocantins, Brazil; 4 Institute of Basic Health Sciences, Federal University of Rio Grande do Sul, Porto Alegre, Brazil; 5 Molecular Biology and Animal Virology Laboratory, State University of Londrina, Londrina, Paraná, Brazil; Eduardo Mondlane University: Universidade Eduardo Mondlane, MOZAMBIQUE

## Abstract

Asymptomatic and underreported individuals remain a source of coronafig disease 2019 (COVID-19) transmission to others. Data on the prevalence and epidemiological factors influencing transmission are fundamental for establishing control measures, especially in vulnerable regions such as the Amazon. This study aimed to determine the point prevalence and active infection of COVID-19 among the population in Araguaína, a Brazilian city located in the Amazon region, analyzed the socioeconomic and behavioral variables of a statistically representative sample of this population using an epidemiological survey, and identify the viral genomic diversity in the region. During the sixth epidemiological week of 2021 (February 8 to 12), samples of 497 inhabitants of the municipality asymptomatic for respiratory syndromes underwent reverse transcription-quantitative polymerase chain reaction and serological tests (immunoglobulin M and immunoglobulin G). A questionnaire collated data on socioeconomic factors, prevention measures, and health status history. The active infection rate was 6.2%, and the prevalence was 13.5% of the study population. Active infection cases were under-reported; each reported positive case represented 14–28 under-reported cases. Lineages P.2, P.1, and B.1.1 were detected. Working from home was a protective factor against the infection, and clinical signs of fever, dry cough, and loss of taste or smell were associated with testing positive (*p* <0.05). A descriptive analysis of the indicators revealed that the entire population was susceptible to the disease. Intensified vaccination strategies are required regardless of socioeconomic factors, health conditions, and preventive measures. Implementation of objective, comprehensive, and efficient management tools to minimize the spread of COVID-19 in this municipality can serve as a model for other regions of Brazil.

## Introduction

According to the Brazilian Ministry of Health 2023 statistics, Coronavirus disease 2019 (COVID-19) has claimed ≥ 700,000 lives in Brazil due to its high transmission rate [1]. In Brazil, besides mortality, COVID-19 has resulted in disability-adjusted life years (DALYs), particularly among socially vulnerable groups of people, and high economic losses, including direct treatment costs and government financial aid. Molecular tests and rapid test kits are used to identify and isolate individuals who test positive for COVID-19 to control the spread of the virus [2, 3]. The identification of asymptomatic and oligosymptomatic patients is challenging. These patients might be diagnosed as having other illnesses, as the symptoms of COVID-19 are very similar to common respiratory infections [4–6].

Testing the largest possible percentage of the population is an effective method for identifying and isolating asymptomatic and oligosymptomatic patients. However, the disease is underreported in Brazil due probably the testing constraint given by increased global demand for tests and the operational limitations of conducting tests in the Brazilian healthcare system [7].

Brazil has poor diagnostic coverage for large-scale COVID-19 testing among the population and is ranked 149^th^ globally, with 296,000 tests per million inhabitants [8, 9]. Lack of testing in asymptomatic individuals is an obstacle that undermines the reliability of the official data on the actual number of cases. Testing is restricted to severe cases, and the monitoring and isolation of patients with mild symptoms and their primary contacts are performed without a confirmed laboratory diagnosis [10].

Regarding asymptomatic infections globally, Oran and Topol [11] reviewed studies that used RT-qPCR for COVID-19 diagnosis and concluded that 40–45% of people infected with severe acute respiratory syndrome coronavirus-2 (SARS-CoV-2) remain asymptomatic and are able to transmit the virus for up to 14 days, indicating the possibility of silent disease spread among the population. Global estimates, however, varied considerably. Al-Sadeq and Nasrallah [12] in a systematic review, observed that the estimate of asymptomatic cases reported in several larger studies (>10,000 participants) varied from 1.2% to 12.9%, however, in studies with smaller samples, the percentage of asymptomatic cases was estimated to be up to 87%.

COVID-19 epidemiological studies primarily rely on analyzing data from digital notification systems in hospitals, clinics, and other healthcare settings [13]. Silveira et al. [14] used a probabilistic population sample to conduct household surveys in Southern Brazil. They used rapid test kits to diagnose COVID-19 probably without sufficient sensitivity for reporting highly accurate data to devise strategic public policies to control the pandemic. These authors noted a significant under-reporting of cases (9 out of 10). Do Prado et al. [7] estimated that underreported cases were approximately 11 times higher than reported cases in the Brazilian population.

At the beginning of the pandemic in Brazil, Hallal et al. [15] conducted a prevalence study and household surveys with residents of large cities in 27 federation units with the help of 57,000 volunteers. They reported the highest prevalence of COVID-19 in the country’s northern region (Amazon). This study found a prevalence of >10% in 11 municipalities of the Amazon region, and a general increase in prevalence over time, indicating that the high prevalence in this region is related to low-income status and limited healthcare services. However, this study did not use significant population sampling of the city, which might have resulted in inaccuracies in the documented results. Fellows et al. [16] revealed that the incidence and mortality rates were higher in the Legal Amazon indigenous people than the national average, with 14% underreporting of cases and 103% of deaths, suggesting that this population is highly susceptible to SARS-CoV-2.

Several non-pharmaceutical measures have been recommended to combat the pandemic [17, 18]. However, there is a need to routinely assess the impact, compliance, and effectiveness of these measures at the population level to propose improvements, adjustments, or suspensions at the appropriate time. The reduction of restrictions on businesses and shared spaces was declared in several Brazilian states in mid-2020 due to economic and political pressures [19, 20].

Because the population of the Amazon region is more vulnerable to the impacts of the pandemic, given the context of geographic accessibility, composition of the population, and reliability of healthcare infrastructure, this study aimed to determine the point prevalence and infection active of SARS-CoV-2 in the asymptomatic population of Araguaína, Tocantins, in the Amazon region. The community’s economic, social, and behavioral variables were analyzed using statistically significant population sampling in an epidemiological study.

## Materials and methods

This cross-sectional study focused on the residents of Araguaína, a municipality in the northern Tocantins State, Brazil. Epi Info^™^ version 7.2.3.1 (CDC, Atlanta, GA, USA, https://www.cdc.gov/epiinfo/) was used to determine the minimum representative sample size, with an estimated population size of 183,381 residents [21]. To ensure the representativeness and statistical significance of the results, an expected frequency of 20%, design effect of 1.4, and confidence limit of 95% were used, and a minimum sample size of 345 was estimated.

An epidemiological survey (S1 File Questionnaire), blood samples for serological tests (immunoglobulin M [IgM] immunoglobulin G [IgG]), and nasopharyngeal swabs for RT-qPCR tests were used to collate data. During the sixth epidemiological week of 2021 (February 8–12), 497 participants voluntarily attended and agreed to participate in the survey. Only participants who self-reported as asymptomatic (for respiratory syndromes) on the day the survey was collected, ≥ 18 years old, and who provided informed consent were included in the study. The study was approved by the Ethics and Research Committee of the Federal University of Tocantins Foundation (CAAE certificates 38298620.3.0000.5519 and 39867820.0.0000.5519). Positive samples of sufficient quality were selected for genome sequencing and phylogenetic analyses. Detailed information about data collection, participant selection, serological testing for COVID-19, RT-qPCR testing for SARS-CoV-2, genome sequencing, and phylogenetic analysis is available in S2 File Materials and Methods (Supporting Information).

Epi Info^™^ version 7.2.3.1 was used to analyze the results of the serological and molecular tests and survey responses. Positive using at least one IgM, IgG, and RT-qPCR test results were used to calculate the point prevalence. For the purposes of data analysis, this study considered active infection only positive results in RT-qPCR [22]. To calculate the underreporting of active cases, data on active cases reported by the Municipal Secretary of Health from Araguaína (MSHA) divided by the actual number of active cases identified in the survey was used. Descriptive and analytical analyses were performed, with the dependent variables being IgM and IgG serological and RT-qPCR results for SARS-CoV-2. Independent variables included socioeconomic data, preventive measures, and health history. Participants with inconclusive or indeterminate results were excluded from the survey analysis. Fisher’s exact test and logistic regression were used for statistical analysis when appropriate. Variables with a significance level <0.25 were selected for multivariate analysis using logistic regression [23]. *P* < 0.05 was considered statistically significant. Odds ratios were used to measure the associated protective or risk factors. Wilson’s test was performed to establish a 95% confidence interval (95% CI) for the descriptive statistical analysis of the data.

## Results

### Positive diagnostic tests for COVID-19, under-reporting, and serological and molecular test analyses

The distribution of the point prevalence of SARS-CoV-2 in the participants is presented in Table 1 (complete data in S1 Dataset).

**Table 1:** Distribution of positive, inconclusive, and indeterminate SARS-CoV-2 test results among the 497 participants from the municipalities of Araguaína, Tocantins, asymptomatic for respiratory syndromes from February 8 to 12, 2021, according to the residence.

Region	Participants		Positive on RT-qPCR		Indeterminate in RT-qPCR		Positive for IgM		Positive for IgG		Inconclusive for IgG		Positive in at least one diagnostic test	
	n	%	n	%	n	%	n	%	n	%	n	%	n	%
**Center**	80	16.13	8	10	0	-	3	3.75	9	11.25	0	-	15	18.75
**North**	181	36.49	11	6.08	2	1.10	5	2.76	14	7.73	0	-	22	12.15
**South**	105	21.17	4	3.81	0	-	4	3.8	8	7.62	0	-	10	9.52
**East**	49	9.88	3	6.12	0	-	3	6.12	5	10.2	2	4.08	10	20.41
**West**	82	16.49	5	6.1	3	3.66	3	3.65	6	7.3	1	1.21	10	12.2
**Total**	**497**	**100**	**31**	**6.24**	**5**	**1.01**	**18**	**3.62**	**42**	**8.45**	**3**	**0.6**	**67**	**13.48**

The RT-qPCR test revealed that 6.24% had an active infectious case (95% CI: 4.28–8.74). Considering the city’s entire population, this percentage corresponds to 11,358 residents infected with the virus between February 8 and 12, 2021.

In the same period, the MSHA reported an average of 555 active cases [24]. Our study stratified the cases (11,358/555) and discovered that each reported positive case represented 20 under-reported cases (95% CI: 14–28) of asymptomatic people in Araguaína.

Eighteen (3.62%; 95% CI 2.30–5.65) participants had recent infections, as indicated by a positive IgM serological test. Of these, nine (1.81%) were simultaneously positive by RT-qPCR, IgG, and IgM tests, whereas the others were not positive by molecular testing (S1 Dataset).

Whether the test was molecular or serological, the detected point prevalence was 13.48% (95% CI: 10.6–16.8) of the participants tested. This percentage corresponded to 24,535 residents who had previous contact with the virus or were still in the viral elimination stage. This observation was made in a representative sampled population almost a year after the pandemic began.

Inconclusive RT-qPCR results (1.01%) and indeterminate results (0.6%) were not used to determine active infection or investigate chronic phase antibodies for calculating the point prevalence of the disease, as shown in Table 1.

### Socioeconomic indicators

Data from 489 participants were analyzed to describe the socioeconomic aspects of the representative population of Araguaína. Participants who presented with inconclusive or indeterminate results were excluded from the descriptive analysis. Table 2 shows the descriptive sample characteristics and results of the multivariate analyses (when appropriate). Table 2 presents the results of the univariate and multivariate analyses, and other descriptive characteristics (ethnicity, pregnancy status, economic relationship between the residents, number of residents, and rooms).

**Table 2:** Socioeconomic characteristics and molecular or serologically positive diagnostic tests for COVID-19 with 489 participants living in Araguaína, Tocantins, who were asymptomatic for respiratory syndromes between February 8 and 12, 2021.

Socioeconomic Indicators	Descriptive				Positivity		Multivariate analysis^a^		
	n	%	95% CI		n	%	Odds Ratio	95% CI	*p*-value
**Gender** *									
Male	222	45.40	41.04	49.83	27	12.16			
Female	267	54.60	50.71	58.96	37	13.86			
Total	489				64				
**Age group** *									
18–19 years	6	1.23	0.43	3.56	0	-			
20–29 years	86	17.58	9.40	33.30	12	13.95			
30–39 years	108	22.08	12.41	39.20	13	12.04			
40–49 years	121	24.73	14.36	42.53	13	10.74			
50–59 years	89	18.19	9.76	34.17	13	14.61			
60–69 years	55	11.24	5.12	25.00	10	18.18			
70–79 years	16	3.26	1.06	10.90	3	18.75			
80 years or more	8	1.62	0.38	7.56	0	-			
Total	489				64				
**Education** *									
No education/illiterate	13	2.66	1.56	4.49	0	-			
Elementary school complete/incomplete	87	17.79	12.00	27.61	18	20.69			
High school complete/incomplete	132	27.00	22.34	32.78	20	15.04			
College education complete/incomplete	252	51.53	44.66	59.17	26	10.32			
Total	489				64				
**Type of aid**									
Did not receive any aid	335	68.51	64.26	72.47	45	13.34			
Received emergency aid from the federal government	125	25.56	21.90	29.61	15	12.00			
Bolsa Família	14	2.86	1.71	4.75	3	21.43			
Other aids (retired, pension)	16	3.27	2.02	5.25	1	6.25			
**Main occupation**									
Student	64	13.09	10.38	16.37	7	10.94			
Active worker	204	41.51	37.23	45.93	32	15.69	1.4706	0.8584–2.5193	0.1603
Home office worker	75	15.34	12.41	18.80	5	6.67	0.4705	0.1811–1.2223	0.1216
Unemployed before the pandemic	41	8.38	6.24	11.18	5	12.20			
Unemployed after the pandemic	30	6.13	4.33	8.62	2	6.67			
Employment contract suspended	3	0.61	0.21	1.79	0	-			
License or vacation	5	1.02	0.44	2.37	2	40.00	4.9585	0.7936–30.9802	0.0868
Business activity suspended	4	0.82	0.32	2.08	0	-			
Businessperson activity	16	3.27	2.02	5.25	3	18.74			
Retired or pensioner	75	15.34	12.41	18.80	11	14.67			
**Activity performed since the beginning of the pandemic**									
Studied or worked outside home	287	58.69	54.28	62.97	37	12.89			
Studied or worked at home office	222	45.40	41.04	49.83	18	8.14	0.4360	0.2421–0.7853	**0.0057**
Did physical activity outside home	237	48.47	44.07	52.89	29	12.13			
Did any physical activity at home	187	38.24	34.04	42.62	21	11.29	0.9116	0.5142–1.6162	0.7515
Did any supermarket shopping	458	93.66	91.14	95.50	59	12.88			
Attended parties or social gatherings	185	37.83	33.64	42.21	24	13.04			
Traveled to other cities	270	55.21	50.78	59.56	34	12.64			
Traveled to another state or country	190	38.85	34.64	43.25	24	12.63			
Visited family or friends	373	76.28	72.31	79.83	49	13.10			
**Means of transportation**									
Public transportation	16	3.27	2.02	5.25	2	12.50			
Own car	255	52.15	47.72	56.54	32	12.60			
Own motorcycle	157	32.11	28.12	36.37	24	15.19			
On foot	19	3.89	2.50	5.99	1	5.26			
Taxi/Uber	13	2.66	1.56	4.49	1	7.69			
Motortaxi	10	2.04	1.11	3.72	1	10.00			
Bicycle	22	4.50	2.99	6.72	4	17.39			
Hitchhiking car/motorcycle	25	5.11	3.49	7.44	2	8.00			

*Descriptive analysis alone.

^a^Performed only for those variables that presented a significant result in the univariate analysis or a significant level < 0.25 using logistic regression.

Among those surveyed, COVID-19 positivity results were very similar between age groups with a slight increase in percentage among people aged 60–79 years (>18%), those with a low level of education (n = 18, 20.69%), and those who received financial aid (*Bolsa Família*) (n = 3, 21.43%).

During the pandemic, most study participants (n = 204, 41.51%) were active on-site workers, whereas 75 (15.34%) worked remotely from their homes. However, specific details about the nature of their jobs were not mentioned. Furthermore, 222 (45.40%) participants who had been working or studying from home since the beginning of the pandemic, showed statistical significance in the multivariate analysis (*p* = 0.0057). Thus, working or studying from home is a protective factor (odds ratio, 0.436 [0.2421–0.7853]) against COVID-19.

Most participants received no financial aid, and those who received financial aid from the “*Bolsa Família*” program showed higher rates of COVID-19 positivity. Further analyses did not reveal any statistical significance. Most participants used their own vehicles (cars or motorcycles) for transport; however, the highest percentage of COVID-19 positivity was observed among those who used bicycles (n = 4, 17.39%), followed by motorcycles (n = 24, 15.19%) as their means of transport. However univariate analysis did not reveal a statistically significant association).

### Preventive measures

In the analysis of preventive measures, most participants expressed a desire to be vaccinated as soon as the vaccine was available. They consistently used masks (n = 296, 60.66%). Of all type of used masks, the majority were made of cloth or tissue (n = 396, 80.98%). Regarding replacing masks, more than half of the interviewees (n = 295, 60.33%) stated that they did so within an interval of less than 6 hours. Approximately 44% of participants strictly adhered to the control measures recommended by the authorities (Table 3). Results of univariate analysis and other descriptive characteristics, such as criteria for replacing cloth masks and places frequently visited by participants.

**Table 3:** Preventive measures were adopted and molecular or serologically positive diagnostic tests for COVID-19 with 489 participants living in Araguaína, Tocantins, who were asymptomatic for respiratory syndromes between February 8 and 12, 2021.

Prevention Measures	Descriptive				Positivity		Multivariate analysis^a^		
	n	%	95% CI		n	%	Odds Ratio	95% CI	*p*-value
**Intention to get vaccinated**									
Does not want to get vaccinated	23	4.70	3.15	6.96	7	30.43			
Wants to get vaccinated	422	86.30	82.97	89.06	49	76.56	0.4913	0.2132–1.1321	0.0952
Do not know	28	5.73	3.99	8.15	7	10.94	1.2500	0.3928–3.9778	0.7055
Depends on other factors	16	3.27	2.02	5.25	1	1.56			
**Frequency of mask use** *									
Always	296	60.66	56.25	64.89	37	12.50			
Often	134	27.46	23.69	31.58	17	12.69			
Sometimes	50	10.25	7.86	13.25	7	14.00			
Rarely	8	1.64	0.83	3.20	3	37.50			
**Mask type** *									
Cloth or tissue	396	80.98	77.27	84.21	52	13.13			
Surgical/disposable	78	15.95	12.97	19.46	12	15.38			
N95	13	2.66	1.56	4.49	0	-			
PFF2	2	0.41	0.11	1.48	0	-			
**Mask usage time** *									
Less than 6 h	295	60.33	55.93	64.57	30	10.17			
7–12 h	65	13.29	10.57	16.59	12	18.46			
13–24 h	76	15.54	12.60	19.02	10	13.16			
1–2 days	31	6.34	4.50	8.86	8	25.81			
3 days or more	22	4.50	2.99	6.72	4	17.39			
**Criteria for replacing the cloth mask** *									
Every 2 h or if it becomes damp	126	25.77	22.09	29.82	12	9.52			
When it is found to be dirty even without visible soiling	231	47.24	42.85	51.67	32	13.85			
Only when it is dirty	29	5.93	4.16	8.39	6	20.69			
When you remember	14	2.86	1.71	4.75	4	28.57			
Do not know/ did not choose	6	1.23	0.56	2.65	0	-			
**Adherence to control measures** ^b^									
Fairly well	216	44.17	39.83	48.60	27	12.50			
More or less	171	34.97	30.87	39.30	25	14.62			
A little	28	5.73	3.99	8.15	4	14.29			
Very little	28	5.73	3.99	8.15	5	17.86			
Stay practically isolated from everyone	46	9.41	7.13	12.32	3	6.52			

*Descriptive analysis alone.

^a^Performed for only those variables that were significant in the univariate analysis or had a significant level < 0.25 using logistic regression.

^b^Hypothesis: The mean number of people testing positive for COVID-19 was lower among those who adhered extensively to control measures.

Most participants, 422 (86.30%) in this study expressed a desire to receive vaccinations; however, some refused or doubted it. When asked for a reason, they stated the following: “did not find the vaccination agent reliable,” “not necessary to vaccinate,” “wait for more studies,” “has no proven efficacy,” and “do not believe in the immunization potential of the vaccine.” Other participants were conditioned to be vaccinated “if the vaccine is from Oxford,” “if it is mandatory,” “if it is not from China,” and “if it is proven to be harmless for breastfeeding”.

Descriptive analysis shows the rate of COVID-19 positivity in the participants who replaced their masks in 1 or 2 days (n = 8, 25.81%) was more than twice as high as those who replaced them within 6 hours (n = 30, 10.17%). Regarding mask replacement criteria, 2.86% of participants replaced the mask only “when they remembered”, and these participants had (n = 4, 28.57%) cases with COVID-19 positivity.

Regarding social distancing measures, the respondents who claimed to have been “practically isolated from everyone” had the lowest (n = 3, 6.52%) rate of cases with COVID-19 positivity, while those who claimed to have “very little isolation” had the highest (n = 5, 17.86%). However, the multivariate analysis did not show significant differences regarding adherence.

### History of health

Table 4 shows the participants’ self-reported health histories. Details of the univariate analysis and other descriptive characteristics (adopted measures when experiencing COVID-19 symptoms, health insurance status, contact with symptomatic COVID-19-positive individuals, cohabitants’ health status, seeking medical attention for reasons unrelated to COVID-19 symptomatology).

**Table 4:** Health history and molecular or serologically positive diagnostic tests for COVID-19 with 489 participants living in Araguaína, Tocantins, asymptomatic for respiratory syndromes between February 8 and 12, 2021.

Health History	Descriptive				Positivity		Multivariate analysis^a^		
	n	%	95% CI		n	%	Odds Ratio	95% CI	*p*-value
**Medication to prevent COVID-19**									
Azithromycin	36	7.36	5.36	10.02	9	25.00	1.7677	0.6571–4.7552	0.2592
Chloroquine	14	2.86	1.71	4.75	4	30.77	1.8618	0.4713–7.3545	0.3752
Ivermectin	144	29.45	25.58	33.64	23	16.08	0.9330	0.4688–1.8571	0.8435
Zinc	75	15.34	12.41	18.80	14	18.67	0.9140	0.3552–2.3518	0.8520
Vitamin D	78	15.95	12.97	19.46	15	19.23	1.3547	0.5647–3.2494	0.4965
Vitamin C	133	27.20	23.44	31.31	24	18.05	1.4013	0.6678–2.9404	0.3723
Prescription drugs	1	0.20	0.04	1.15	0	-			
Homeopathic medicine	122	24.95	21.32	28.97	17	13.83			
Other medicines	30	6.13	4.33	8.62	3	10.00			
No medication	213	43.56	39.23	47.99	21	9.86			
**Symptoms since the start of the pandemic**									
Fever	123	25.15	21.51	29.18	28	22.76	1.9857	1.0348–3.8107	**0.0391**
Dry cough	151	30.88	26.95	35.11	32	21.19	2.0371	1.0916–3.8015	**0.0254**
Cough with phlegm	102	20.86	17.49	24.68	16	15.69	0.7171	0.3542–1.4518	0.3555
Sore throat	187	38.24	34.04	42.62	27	14.44			
Headache	249	50.92	46.50	55.33	36	14.46	0.7516	0.3920–1.4412	0.3900
Stuffy or runny nose	205	41.92	37.63	46.34	35	17.07	1.6432	0.8822–3.0606	0.1176
Diarrhea	99	20.25	16.92	24.03	16	16.16	1.1892	0.6000–2.3573	0.6196
Loss of smell or taste	71	14.52	11.67	17.92	21	29.58	3.1151	1.5325–6.3319	**0.0017**
Difficulty breathing	77	15.75	12.79	19.24	15	19.48	1.3839	0.6354–3.0141	0.4132
Fatigue (tiredness)	145	29.65	25.78	33.85	22	15.17	0.5756	0.2709–1.2233	0.1510
Pain in the eyes	109	22.29	18.83	26.19	18	16.51	0.8004	0.3948–1.6225	0.5368
Chest pain	69	14.11	11.30	17.48	11	15.94			
Muscle pain (myalgia)	171	34.97	30.87	39.30	24	14.04			
Nausea (motion sickness)	75	15.34	12.41	18.80	12	16.00	0.8762	0.3967–1.9354	0.7438
No symptoms	126	25.77	22.09	29.82	5	3.97			
**Sought medical attention, if symptomatic** *									
Yes	131	26.79	23.06	30.88	29	22.14			
No	231	47.24	42.85	51.67	30	12.99			
Do not remember	1	0.20	0.04	1.15	0	-			
**Action is taken when experiencing symptoms** *									
Sought medical attention	119	24.34	20.74	28.33	27	22.69			
Stayed home and did not take medication	29	5.93	4.16	8.39	3	10.34			
Stayed home and performed self-medication	116	23.72	20.17	27.69	19	16.38			
Stayed home and took home remedies	55	11.25	8.74	14.36	8	14.55			
Stayed home and called the COVID-19 hotline	1	0.20	0.04	1.15	0	-			
Took self-medication and continued with normal activities	38	7.77	5.71	10.49	5	13.16			
Did not take medication and continued with normal activities	18	3.68	2.34	5.74	0	-			
Received a visit from the health professional	4	0.82	0.32	2.08	1	25.00			
**Tested for COVID-19** *									
Yes	187	38.24	34.04	42.62	35	18.82			
No	302	61.76	57.38	65.96	29	9.57			
**Possible place where you contracted the disease** * ^,^ ^b^									
At work	89	18.20	15.03	21.86	17	19.10			
At home with family	105	21.47	18.06	25.33	21	20.00			
At home with relatives	17	3.48	2.18	5.50	2	11.76			
Parties/gatherings	14	2.86	1.71	4.75	2	14.29			
Supermarket	23	4.70	3.15	6.96	4	17.39			
School/courses	1	0.20	0.04	1.15	0	-			
Church/churches	0	-	-	-	0	-			
Other places	92	18.81	15.60	22.52	8	8.70			
**Participant positive for COVID-19, in isolation, went to:** *									
Supermarket	5	1.02	0.44	2.37					
Pharmacy	5	1.02	0.44	2.37					
Healthcare service	4	0.82	0.32	2.08					
Solve work demands	2	0.41	0.11	1.48					
Visit relatives	3	0.61	0.21	1.79					
Help someone	0	-	-	-					
Country house or farm	5	1.02	0.44	2.37					
Did not go out during isolation	27	5.52	3.82	7.91					
**Comorbidities** *									
Hypertension	110	22.49	19.02	26.40	17	15.45			
Diabetes	43	8.79	6.59	11.64	5	11.63			
Asthma or bronchitis	34	6.95	5.02	9.56	5	14.71			
Chronic kidney disease	14	2.86	1.71	4.75	3	21.43			
Chronic heart disease	26	5.32	3.65	7.68	3	11.54			
Chronic lung disease	10	2.04	1.11	3.72	1	10.00			
Immunosuppression	15	3.07	1.87	5.00	5	33.33			
Cancer	4	0.82	0.32	2.08	0	-			
High-risk pregnancy	3	0.61	0.21	1.79	0	-			
Other chronic diseases	130	26.58	22.86	30.67	18	13.85			
**Disease developed during the pandemic** *									
Diabetes	5	1.02	0.44	2.37	0	-			
Decompensated diabetes	8	1.64	0.83	3.19	1	12.50			
Hypertension	11	2.25	1.26	3.98	2	18.18			
High Cholesterol	41	8.38	6.24	11.18	3	7.32			
Cancer	1	0.20	0.04	1.15	0	-			
Depression	24	4.91	3.32	7.20	3	12.50			
Anxiety	132	26.99	23.25	31.10	16	12.12			
Other psychological disorder	19	3.89	2.50	5.99	3	15.79			
Other illness	37	7.57	5.54	10.26	4	10.81			

* Descriptive analysis only.

^a^ Only those variables that showed a significant result in the univariate analysis or significant level < 0.25 using logistic regression.

^b^ The more appropriate term is “Possible site where you contracted the infection.”

As this is a new disease with a high transmission rate and uncertain outcomes, combined with the unreliability of health services in caring for the sick, 213 (43.56%) individuals stated that they had not administered any medication to prevent COVID-19. However, the rest administered drugs or home remedies with no proven efficacy. Ivermectin (n = 144, 29.24%), vitamin C (n = 133, 27.20%), and home remedies (n = 122, 24.95%) were used by the participants to prevent COVID-19. Although marketing was controlled, the participants also administered azithromycin (n = 36, 7.36%) and hydroxychloroquine/chloroquine (n = 14, 2.86%). Since the mean number of people that tested positive for COVID-19 was the same among those who did or did not administer these medications (Table 4), showing no statistical significance, the lack of efficacy of the administered medication should be considered.

The volunteers were asked whether they had experienced COVID-19-associated symptoms at any moment since the beginning of the pandemic. Fever, dry cough, and loss of taste and smell showed a significant difference in the multivariate analysis (Table 4). It showed that an individual with COVID-19 was 1.98 times more likely to have a fever, 2.04 times more likely to have a dry cough, and 3.11 times more likely to have a loss of smell or taste than a person with a negative COVID-19 result.

However, 126 (25.77%) participants report had no symptoms since the beginning of the pandemic, and five (3.97%) tested positive using at least one diagnostic test.

Regarding those who stated that they had ever presented any symptoms, since the beginning of the pandemic, n = 38 (7.7%) of participants did not seek medical care, and five (13.16%) were positive, that self-medicated and continued to perform their usual activities, indicating symptomatic carriers who were not isolated from the community. These five positives represented 1,862 individuals’ absolute number in the city. Table 4 shows that the rate of positive cases was higher among those who claimed, “fear of getting infected in the health service” (n = 5, 18.52%) followed by those who “had only mild symptoms” (n = 8, 16.67%).

When participants revealed a positive COVID-19 test result predating this study, we investigated their adherence to the health authorities’ mandate for home isolation. Among the respondents, five admitted to visiting the supermarket, five to drugstores, five to farms, and four to health services. Additionally, three participants acknowledged visiting relatives. This behavior can have severe implications for the transmission of the virus within the population. A high number of positive cases were recorded among those who had immunosuppression five (33.33%), and chronic kidney disease three (21.43%), followed by those with hypertension 17 (15.45%), and asthma or bronchitis five (14.71%). Depression and other psychological disorders have been reported, with the rate of COVID-19 positivity in individuals ranging from 12.12% to 15.79%.

### Genome sequencing and phylogenetic analysis

**Fig 1:**
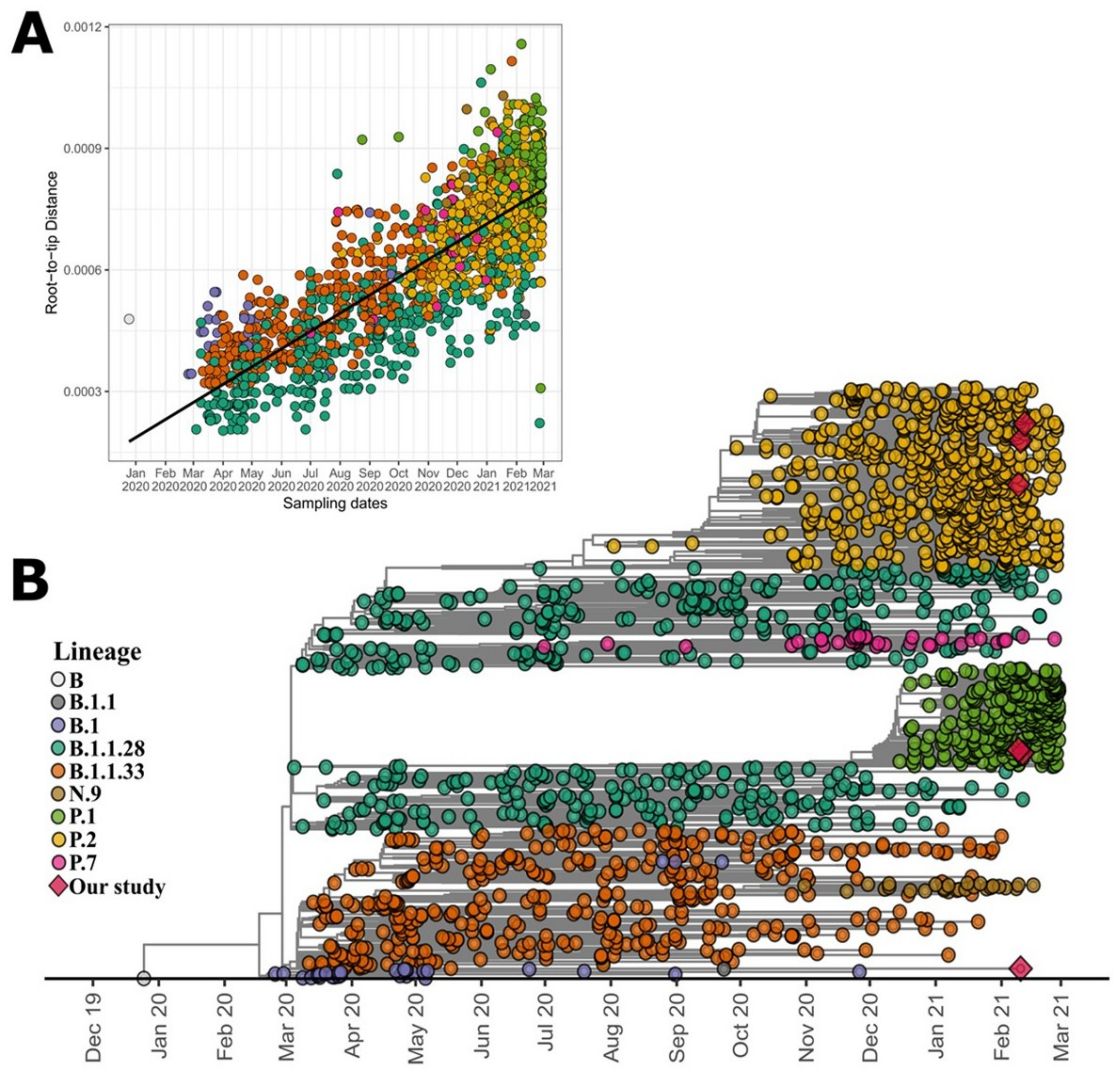
Phylogenetic analysis of SARS-CoV-2 genome sequences in Araguaína, Brazil. **(A)** Root-to-tip regression of genetic distances and sampling dates for the 1,875 SARS-CoV-2 genomes (R^2^ = 0.69; correlation coefficient = 0.83). **(B)** Time-resolved maximum-likelihood tree of 1,875 SARS-CoV-2 genomes. Tips are colored according to the PANGO lineages and red diamonds represent Araguaína genomes.

Regarding the SARS-CoV-2 genomic variability, three of the six sequenced, belonged to lineage P.2, two to lineage P.1, and one to lineage B.1.1. Root-to-tip regression analysis between genetic divergence and sampling dates using the best-fitting, root showed a strong correlation (R2 = 0.69; correlation coefficient = 0.83). The PANGO lineages were assigned to six sequenced genomes from the Araguaína Municipality, according to the time-resolved phylogenetic tree. TreeTime estimated a time to the most recent common ancestor (tMRCA) of November 25, 2019, with an estimated substitution rate of 8.0×10^−4^. The phylogenetic tree demonstrated that SARS-CoV-2 genomes from the early stages of the pandemic in Brazil, mainly to B.1.1.28 and B.1.1.33 lineages (Fig 1).

## Discussion

This study demonstrated the underreporting of active COVID-19 cases in Araguaína, Brazil. Underreporting may be related to asymptomatic disease cases, delays in test results, a lack of tests, or difficulties in operationalization [7, 25]. Asymptomatic infection may be related to several factors such as age, demographics, viral load, magnitude, and immune strength [26]. Without regular testing of individuals, it will be difficult to identify positive cases, especially asymptomatic. Hence, regular testing is essential to reduce underreporting and control the movement of infected individuals. Espenhain et al. [27], who estimated the under-reporting of COVID-19 diagnosed by RT-qPCR, demonstrated a reduction in under-reporting over time, from five underreported cases in spring with just one in early December, which was undoubtedly due to an increase in testing from 15 to 50/1000 inhabitants.

Most prevalence studies have focused on hospitalized individuals, healthcare workers, and immunosuppressed individuals, limiting their representativeness of the general population [28–31]. Our study, with a significant sample of the general population, identified a prevalence of 13.48%, which was similar to the prevalence calculated by the MSHA since the first case in the municipality, with confirmed COVID-19 diagnosis in 20,380 individuals, which corresponds to 11.20% of the population [24], a percentage that is similar to the results of this study. However, the prevalence may have been underestimated if the IgG anti-SARS-CoV-2 antibody titer, which depends on the intensity of the immune response and disease severity, had decreased beyond detectable limits by the time of the diagnostic evaluation (approximately 6 months) [32, 33]. Therefore, the participants who were COVID-19-positive, but were evaluated after the time for detecting antibodies had elapsed may not have been identified in these studies. This possibility was confirmed by our analysis, as 41 (8.25%) participants had a previous diagnosis of COVID-19, and 11 (2.21%) maintained detectable IgG titers at the time of our study. Furthermore, Espenhain et al. [27] found that 5% of the positive participants did not develop IgG antibodies 14 days after RT-qPCR confirmation, attributing it to reduced immunity or mild or asymptomatic infection that did not trigger a detectable antibody response. In a review, Rostami et al. [34] reported the pooled seroprevalence of the studies. Countries with the highest proportions were Iran (22.1%), Sweden (15.02%), Chile (10.7%), Switzerland (7.9%), Italy (7.27%), South Korea (7.5%), Spain (5.0%), and the USA (4.4%), and the overall proportion observed globally was 3.38%.

Our search yielded inconclusive and indeterminate results. This was due to the limitation of the study (cross-sectional) that did not allow to carry out new collections of the participants, and the diagnostic tests used to make it possible to deliver indeterminate and inconclusive results. For Das et al. [35], inconclusive RT-qPCR results possibly related to RNA extraction failure, the presence of PCR inhibitors in the sample, the mutation of the virus in the target region, improper or inadequate sample, and timing of the sample in relation to the clinical course of the disease. We believe that the variations in the research results may be attributed to participants who experienced either recent or late-stage infections, wherein they might not have developed a sufficient quantity of antibodies or nucleic acids specific to the virus. To understand the behavior of a disease in a population, it is essential to know its main epidemiological characteristics. Our study identified similar positivity for COVID-19 among age groups, with a slight increase in the age group of 60 to 79 years. These results differed from those reported by the municipal epidemiological surveillance [36], in which 6.1% of the reported cases were between 60 and 69 years, and 3.1% were between 70 and 79 years, with the highest mortality rates recorded in these age groups being 24.1% and 27.8%, respectively. This finding is important because contrary to the official data which reports lower positivity among older adults, our study in comparison, demonstrated high positivity rates in the older population, corroborating possibly higher mortality rate recorded in these age groups. A higher prevalence among older adults may result in higher mortality due to the frequent presence of comorbidities [37]. Other studies recorded a lower prevalence among older adults. For instance, the study by Pollan et al. [38] in Spain detected a seroprevalence of 4.5% in the population aged ≥65 years, and a systematic review by Rostami et al. [34] estimated a 2.57% seroprevalence of the global population aged ≥65 years.

Education can help understand the importance of compliance with preventive measures. Our study demonstrated that a higher proportion of COVID-19 cases are related to a low education level. Rezende et al. [39] reported that adults with a lower level of education were twice as likely as college students to have COVID-19. These findings demonstrate that informal language should be used to disseminate information about the disease so that everyone, regardless of educational level, can understand it.

To minimize the socioeconomic impact of the pandemic among vulnerable populations, Brazil granted financial aid to these populations, which was also provided by Spain and the USA to their own populations [40]. Despite the availability of financial aid, our study showed that COVID-19 positivity rates were similar between those who were similar among those who did and did not receive any type of aid, which indicates that even with aid, people could continue to be at risk, whether they were working in financial activities or practicing essential activities.

Several restrictions were imposed by the local government, such as suspending physical attendance at class and allowing only virtual attendance; workers with comorbidities were only permitted to work from home; social events, parties, and get-togethers were prohibited; and essential shopping was limited to essential establishments, such as supermarkets, with restricted entry along with restrictions on leisure travel [41, 42]. Our investigation showed that working or studying from home was a protective factor against COVID-19, likely due to the reduce temporal exposure in community settings, which consequently lowered virus transmission. These findings reinforce the importance of restricting activities and promoting remote studies as preventive measure against COVID-19 whenever possible [43, 44].

Public transportation is highly sensitive to the disruptions and reflections of the pandemic because of the collective nature of its mobility [45]. The type of transportation used was evaluated based on the turnover of passengers, contact of passengers with surfaces that are often difficult to sanitize, and proximity between individuals. Medlock et al. [46] had previously reported that public transport is highly correlated with an increased likelihood of transmission, possibly due to crowding and higher movement of people. However, our study did not detect an association between COVID-19 and the use of different types of transportation. This may be because only a small proportion of our study population (n = 16, 2.02%) used public transport.

Vaccine development aims to generate an immune response without causing disease [47]. However, doubts about the safety of the immunogen, the efficiency of the vaccine, the short time of vaccine development, fear of injection, and the need for more information about the vaccine have been reported as reasons for hesitation in taking the vaccine [48]. Our survey detected a certain level of vaccine anxiety among respondents, demonstrating widespread queries about the credibility of immunizing agents and their social impact. In June 2020, Khubchandani et al. [49] surveyed the intent to vaccinate in the USA. They found that 52% of respondents said, “very likely,” while 7% said, “definitely not”. Participants with lower education and income were more likely to not want to be vaccinated (i.e., vaccine hesitancy), and similar results were obtained by Edwards et al. [50] in Australia. In our study, which was conducted before vaccines were available, many expressed their intention to be vaccinated; however, the proportion that was uncertain about immunization (participants who responded “don´t know” and “depends on other factors” in the survey) was >9%, indicating that the authorities needed to intensify promotions about the reliability of vaccines as a safe and important tool to contain the pandemic. Authorities must counter fake news that undermines vaccination drives.

Pending the development and availability of vaccines against COVID-19, the population requires other preventive measures that were widely publicized in the media [51]. One such measure is the use of masks (fabric, disposable, PFF2, or N95), which are required for people’s movement through public environments. These masks were effective in minimizing the transmission of COVID-19 [52]. Masks such as PFF2 and N95 provide greater protection to the respiratory tract than common disposable or fabric masks [17, 53, 54]. Due to low availability and high cost compared to others, only 5.72% of the population used these masks. However, the COVID-19 test results of these users, were negative, highlighting a higher efficiency of these masks than other types.

Another important indicator of success in preventing COVID-19 is the periodic replacement of cloth masks. Ideally, masks should be replaced every 2 or 3 h for optimal efficiency or if wet [54]. Our study indicated that noncompliance with these guidelines could contribute to a greater risk of infection. Mitze et al. [52] reported that after the local government imposed the mandatory use of masks, there was a 75% reduction in the number of COVID-19 cases and up to 90% reduction of cases in the age group of >60 years, indicating the importance of mask use that overshadow the effects of other prevention measures.

High adherence to control and prevention measures of the population may be the key to containing the spread of the disease. At the beginning of the pandemic, Barros et al. [20] researched the adherence to social distancing measures in populations of cities in the state of Rio Grande do Sul, Brazil. They reported that 41.1% of respondents claimed to have followed “enough” of these measures and that individuals aged 25–59 years reported less isolation. This was unlike the >60-year age group, which appeared to be more protected, as 80% claimed to be isolated or practiced extensive social isolation. Jones et al. [18] reinforced that the minimum distance between asymptomatic individuals should be 2 m, considering ventilation, occupation, and exposure time. They suggested that physical distancing rules would be more effective if they reflected gradual levels of risk. In our survey, almost half of the interviewees admitted that they had complied very well with the control measures recommended by the authorities. In contrast, one-third had complied moderately, showing that the authorities still need to take a different approach to enable greater acceptance of such measures by the population.

Health conditions are intrinsic to all individuals. Individuals who did not have any pre-existing conditions prior to COVID-19 infection have an advantage over those who have some physiological disorders or other pre-existing debilitating diseases that predisposes them to an unfavorable outcome [6], demonstrating the presence of anxiety in the population about administering medications to prevent or treat the disease, even though most studies on drugs are ongoing, and scientific proof from all phases of research is yet unavailable [55–57].

This study showed that a large portion of the population believed that the administration of some medications could prevent COVID-19, probably because of the questionable information that the drugs would have COVID-19 preventive efficacy [58]. The administration of drugs to prevent COVID-19 may have generated a false sense of protection and reduced adherence to proven effective non-pharmaceutical preventive measures. Yin et al. [59] found that administration of antibiotics in the early stages of COVID-19 without bacterial infection was associated with worse disease progression, and azithromycin was unrelated to better clinical outcomes.

In addition to the asymptomatic form, COVID-19 can present various symptoms [59]. Fever, dry cough, and loss of taste and smell showed a significant difference in the multivariate analysis (Table 4), indicating that these symptoms are associated with COVID-19. Espenhain et al. [27] detected a strong correlation between loss of smell and taste and seropositivity. Other studies have indicated a high prevalence of these symptoms [45, 60] which reinforces the clinical characteristics of the disease.

Our record of asymptomatic individuals (3.97%) differed from other estimates [26, 61]. Most likely because of the long evaluation period (11 months), the decline in IgG antibodies after 6 months [32] may not identify asymptomatic individuals in the early stages of the pandemic. However, studies on neutralizing antibodies demonstrate the antibody sustaining duration more accurately [62]. An epidemiological and serological survey study by Espenhain et al. [27] reported that 27% of the respondents had no symptoms associated with COVID-19. However, it was estimated that a much higher percentage had the disease in an asymptomatic form (41% of positive patients) since younger participants were more likely not to answer the survey questionnaire and present the asymptomatic form. Johansson et al. [61] evaluated asymptomatic COVID-19 patients during various infection periods and estimated that more than half of the transmissions occurred in these patients, suggesting that the use of masks, hand sanitization, social distancing, and strategic testing of people can reduce this transmission.

We recorded the confirmation of symptomatic individuals who did not seek medical attention. The search for medical care is essential to follow the clinical evolution and, above all, to isolate and track potentially infected individuals, which is fundamental to controlling disease spread. These data reinforce the high underreporting rate despite the intense media publicity on the need for medical care.

Mass population testing is important for identifying symptomatic and asymptomatic individuals, and the resulting data will help understand the disease’s spread. Over one-third of our participants said they had undergone at least one COVID-19 test, with considerable positivity, an expected observation since the test is generally performed by those who have doubts about their health status or participants in screening programs [63].

Presupposing the place or environment of possible infection is important for intensifying educational campaigns that reduce contagion. To this end, the volunteers were asked where they believed they had contracted the infection, which manifested through the symptoms presented. The places with the highest suspicion of contracting the infection coincided with the frequency of COVID-19 positivity. Participants frequently reported being “at home with the family” and “at work,” indicating that they may have observed other people with symptoms or had knowledge of their health status. Furthermore, 61.69% of the respondents said they had had contact with a person known to be COVID-19 positive. In a telephone survey of adult inpatients and outpatients with positive tests for SARS-CoV-2 conducted by Tenforde et al. [60], participants reported close contact with family members (45%) and coworkers (37%) who were positive, highlighting the need for isolation of infected persons, tracking, and investigation of cases. They reported that two-thirds of the participants were employed; however, only 17% were able to telecommute, highlighting the need to maintain conditions that offer protective measures to workers.

This study revealed that some individuals known to be COVID-19-positive violated their home isolation, which was mandated by health authorities. Violation of home isolation is worrisome because such COVID-19-positive individuals are a source of infection for others, especially in high-traffic places, such as supermarkets. Infringement constitutes critical conduct and is defined as a crime in the Brazilian Penal Code, Article 268: “Infringing determination of the public power, intended to prevent the introduction or spread of contagious disease” [64]. Isolation and quarantine measures must be well-structured to guarantee that an individual’s financials receive compensation for missed workdays, psychological needs, food, and medical support. Frequent violators should be legally persecuted [65]. Seeking health services may be considered the only exception for leaving isolation. Although the questionnaire did not cover the reason for seeking healthcare services, this place is already prepared to receive potentially infected individuals.

Comorbidity is a risk factor for aggravation in COVID-19-positive patients, especially hypertension, diabetes, and obesity [66]. This study demonstrates a considerable frequency of positive results among patients with comorbidity. Immunosuppression status was not associated with an increased risk of developing COVID-19 in a meta-analysis by Tassone et al. [67]; however, most participants were hospitalized COVID-19 patients, which differs from the present study population. A high percentage of the population with hypertension and diabetes may present with severe disease that would overcrowd inpatient medical services and likely overload the healthcare system. Rezende et al. [39] estimated that between one-third and one-half of the Brazilian population, approximately 86 million people, were at risk of severe COVID-19 due to comorbidity.

The COVID-19 pandemic has raised additional health concerns since the beginning of 2020. Therefore, the participants were asked about the development of other illnesses, mainly related to psychological and emotional causes. At least one-quarter of the participants claimed that they had developed anxiety disorders. With the advent of this infectious event, symptoms of diseases, such as anxiety, depression, insomnia, and acute stress were observed in the population, probably among those with confirmed or suspected COVID-19 and their relatives, people at occupational risk of the disease, individuals who underwent quarantine, and those who had not yet returned to work. These findings indicate severe emotional distress, which should be prevented and treated during disease outbreaks [68]. The development of hypercholesterolemia (7.32%) may be related to reduced physical activity caused by the closure of open areas, parks, gyms, and sports centers and due to the trend that more time indoors increased the frequency of unbalanced eating. Similar results were observed by BinDhim et al. [69], who had followed the quarterly population data since the beginning of the pandemic.

Genomic surveillance of SARS-CoV-2 is essential for monitoring its spread and evolution. The samples from this study allowed us to identify, for the first time in early February 2021, the circulation of the P1 variant originating in the Amazon region of the state of Tocantins, even before the first official announcement by the State Department of Health at the end of February, highlighting the importance of genomic surveillance of the virus. Zeizer et al. [70] reported that B.1.1.28 and B.1.1.33 lineages were the most prevalent in Brazil during the first COVID-19 wave. However, during the second wave, these two lineages were completely substituted by the P.2 (Zeta) and P.1 (Gamma) lineages. Souza et al. [71] analyzed the SARS-CoV-2 genomes deposited in Global Initiative on Sharing All Influenza Data (GISAID) until June 2021 and confirmed the high prevalence of P.2 and P.1 lineages in Brazil and its northern region.

This study had some limitations. The data were self-reported and subject to recall bias, as there was a time gap between the onset of the pandemic and the day of the interview (approximately 11 months). During the interviews, the participants were informed about confidentiality, and the interviewers were trained to make people as comfortable as possible. However, the participants’ responses were subject to their own truthfulness, as they may provide untrue responses to hide their misconduct. Another limitation is that our study included only those aged ≥18 years. Moreover, our representative sample had a substantial proportion of participants aged >60 years, which might be because retired or inactive individuals were available to participate in the survey. Furthermore, residents may have opted to choose the oldest residents for this survey to learn about their health status.

## Conclusion

In conclusion, this study identified a high underreporting of active SARS-CoV-2 infections in a statistically significant population of the Araguaína municipality. The point prevalence was comparable to that identified by the MSHA, potentially attributable to the drop in detectable chronic-phase antibodies. Multivariate analyses identified that working or studying from home were protective factors against COVID-19 infection. Moreover, symptoms, such as fever, dry cough, and loss of taste and smell were primary clinical manifestations associated with disease positivity. We detected the first circulation of the P.1 lineage in Tocantins, Brazil. Descriptive data showed regardless of socioeconomic conditions, adoption of preventive measures, and health history, the entire population was found to be vulnerable to contracting the infection. This indicates the need for intensified vaccination campaigns and the development of centralized, efficient, and directed health management tools to help reduce the spread of COVID-19 in Araguaína and the entire country. This research can help to understand some epidemiological and behavioral aspects of the population facing COVID-19.

## Supporting information

S1 File Questionnaire. (PDF)

S2 File Materials and methods. (PDF)

S1 Dataset Results of the diagnostic tests. (XLSX)
