# Supplementary material for: Prevalence, under-reporting, and epidemiological surveillance of COVID-19 in the Araguaína City of Brazil
Source: PLoS One. 2024 Jun 5;19(6):e0300191. doi: 10.1371/journal.pone.0300191 (PMC11152283; doi:10.1371/journal.pone.0300191)
Supplement: S1 File — (PDF) [file pone.0300191.s001.pdf]

**S1 File Questionnaire**

| **Nº** |
|---|

**COVID-19 EPIDEMIOLOGICAL SURVEY**

Filled date: ___/___/___ Applicator name: __________________________

**1. PERSONAL INFORMATION**

Applicator use RG data

**1.1** - Participant name ______________________________________________________________________

**1.2** – CPF _________________________________________ RG: _____________________ Issuing Agency:______

**1.3** – Phone _____________________________________

**1.4** - Date of birth ____/____/____

**1.5** – Gender: ☐ Male ☐Female

**1.6** - Mother's name: ____________________________________________________________________________

**1.7** – Address (Street/Av, nº, Qd/Lt) _______________________________________________________________

District ___________________________________________ City: __________________________________

**1.7.1** - Complement: ☐House ☐ Apartment

**1.8** - How do you identify yourself in terms of color and race? I will read the options:

☐ White ☐ Yellow

☐ Black ☐ Indigenous? Which ethnicity___________________________________________

☐ Brown

**1.9** - Are you pregnant?

☐ No

☐ Not know

☐ Not applicable

☐ Yes

- ☐1º trimester
- ☐2º trimestrer
- ☐3º trimestrer
- ☐Gestational age ignored

**1.10** - Did you study until what grade? (complete/incomplete) (APPLICATOR let the person answer)

☐Schooling/illiterate

☐1ª at 4ª INCOMPLETE Elementary School grade

☐1ª at 4ª COMPLETE Elementary School grade

☐5ª at 8ª INCOMPLETE Elementary School grade

☐COMPLETE Elementary School grade

☐INCOMPLETE Elementary School grade 2º cycle (6ª at 9ª série)

☐ INCOMPLETE high school (1º at 3º ano)

☐ COMPLETE High school

☐ INCOMPLETE College

☐ COMPLETE College

☐ Ignored/blank

☐ Não Not applicable

**1.11** - How many people live with you?

☐No one

☐Nº ____, inform some data

| Name | Gender M /F | Age (years) | Kinship | Tested COVID19? Y/Ñ/DÑ | Which test? P (PCR) S (Sorol) F (Fast) | Result. +/- Inconcl Ignored |
|---|---|---|---|---|---|---|
| | | | | | | |
| | | | | | | |
| | | | | | | |
| | | | | | | |
| | | | | | | |
| | | | | | | |

**1.12** - What is your economic relationship with the people you live in? I'll read the options:

☐You are the head of the family

☐You are kept by the family

☐You help support the family

☐You share rent

**1.13** - How many rooms are there in the house you live in, not counting balconies, open area, material storage?
Nº ________________

**1.14** - Have you received or receive any type of financial assistance? I'll read the options:

☐ Did not receive or do not receive
☐ Bolsa família
☐ Federal Government Emergency Assistance
☐ Another help, which? ______________________________

**1.15** - About your occupation, have you been like during the pandemic? I'll read the options:

☐ Student
☐ Active worker
☐ Home office worker
☐ Unemployed before the pandemic
☐ Unemployed after the pandemic
☐ Employment Contract Suspended
☐ License or vacation
☐ Business activity suspended
☐ Businessperson in activity
☐ Retired or pensioner
☐ Ignored

**1.16** - What is your **current occupation**?____________________________________________________________

**1.17** - What activities below did you carry out during the pandemic? You can say YES in several options.:

☐ Studied or worked outside the home
☐ Studied or worked at home office
☐ Did physical activity outside home
☐ Did any physical activity at home
☐ Did any supermarket shopping
☐ Had parties or social gatherings
☐ Had parties or social gatherings
☐ Traveled to another state or country
☐ Visited family or friends

## 2. PREVENTION MEASURES

**2.1** - When the vaccine is available for your age group/category, will you be vaccinated? (APPLICATOR lets the person respond).

☐ Yes
☐ No? Why? _____________________________________________
☐ Do not know
☐ Depends? From what?_________________________ ___________________

**2.2** – How often have you used the mask since the beginning of the pandemic? I'll read the options::

☐ Always ☐ Often ☐ Sometimes ☐ Rarely ☐ Never

**2.3** - In which situations do you NOT use the mask? I'll read the options and you can choose more than one:

☐ In the home of relatives and/or friends
☐ Church and/or Worship
☐ At parties and/or get-togethers
☐ During outdoor or outdoor sports practice
☐ Another location:_________________________

**2.4** - If you wear a mask, which type do you use the most? I'll read the options:

☐ Woven, cloth
☐ Surgical/disposable
☐ N95
☐ PFF2

**2.5** – How long do you use the same mask before changing/discarding them? I will read the options:

☐ Least 6 hours
☐ 7-12 hours
☐ 13-24 hours
☐ 1-2 days
☐ 3 days or more

APPLICATOR, If you marked CLOTH OR WOVEN in item 2.4, ask the following question, if NO, go to 2.7

**2.6** - If you use the cloth mask more, how often do you change or replace it? I'll read the options:

☐ Every two hours or if it becomes damp
☐ When it is found to be dirty even without visible soiling
☐ Only when it is dirty
☐ When you remember
☐ Do not know/ did not choose

**2.7** – During the pandemic, in which situations did you use alcohol gel? I'll read the options:

☐ After greeting someone with a touch

☐ Before entering commercial establishments
☐ Every time you see the available alcohol bottle
☐ Every time when you touch a surface that you don't know is sanitized
☐ After using bank card machine

**2.8** – "Social distancing measures aim to reduce the spread of diseases such as Coronavirus. The attitude of staying at home and avoiding contact with other people is being guided by several health authorities. In addition to this measure, they are also keeping a distance of 1.5m from other people, wearing masks, leaving the house only for essential activities such as supermarkets, pharmacies, hospitals, avoiding NON-essential activities such as parties, socializing, visits, beaches and tours."
How do you assess your degree of adherence to these measures? I'll read the options: (how much you're managing to do)

☐ Very little
☐ Little
☐ More or less (average)
☐ Quite
☐ It is practically isolated from everyone

**2.9** – When you leave home, what places do you go to? I'll read the options:

☐ Work ☐ Farm or ranch
☐ School/college/course ☐ Church or cult
☐ Gym/Physical activity ☐ Fairs
☐ Supermarket ☐ Department store
☐ Drugstore ☐ Relatives' home
☐ Other places:___________________________________________________________________________

**2.10** - What transport do you use for getting around? I'll read the options::

☐ Public transportation ☐ Mototaxi
☐ Own car ☐ Bicycle
☐ Own motorcycle ☐ Hitchhiking car/motorcycle
☐ On foot ☐ Other _________________________
☐ Taxi/Uber/Transport application

## 3. HEALTH HISTORY

**3.1** – Have you taken any medication or anything to prevent COVID19?

☐ No ☐ Yes, which medicine?
☐ Azithromycin ☐ Chloroquine ☐ Ivermectin ☐ Zinc ☐ Vitamin D ☐ Vitamin C
☐ Prescription drugs at Health Post/Basic Health Unit
☐ Homemade medicines, which? ____________________________________________________
☐ Another one, which one?

**3.2** - Have you had contact with anyone who was sick and tested positive for COVID19?

☐ No ☐ Do not know ☐ Yes, What kinship and/or relationship?________________________________

**3.3** - Since the beginning of the pandemic, have you had any of these signs or symptoms related to COVID19? I'll read the options and you say YES or NO:

| | Yes | No |
|---|---|---|
| Fever | | |
| Dry cough | | |
| Cough with phlegm | | |
| Sore throat | | |
| Headache | | |
| Stuffy or runny nose | | |
| Diarrhea | | |
| Loss of smell or taste | | |

| | Yes | No |
|---|---|---|
| Difficulty breathing | | |
| Fatigue (tiredness) | | |
| Pain in the eyes | | |
| Chest pain | | |
| Muscle pain | | |
| Nausea (motion sickness) | | |
| Other:_____________________ | | |

<u>APPLICATOR, If you presented ANY symptoms above, ask the next question, if NO (no symptoms) go to 3.11</u>

**3.4** – How long has it been more or less since you had these signs or symptoms? ___________________

**3.5** – And did you seek care at any health facility?

☐ No (APPLICATOR ASK THE NEXT QUESTION AND GO TO 3.10)
☐ Doesn't remember (APPLICATOR GO TO 3.10)
☐ Yes, where?

| | | Name of the health facility? |
|---|---|---|
| | Health Post/Basic Health Unit | |
| | SUS/UPA emergency room | |
| | Private emergency room | |
| | SUS Hospital | |
| | Private hospital | |
| | Private practice | |
| | Drugstore | |
| | other health service | |

APPLICATOR, If you checked the option above NO, read the questions below, otherwise go to 3.6

**3.5.1** If you felt these symptoms, why not seek medical attention?
☐ Because the recommendation was to stay at home and only seek medical attention when signs and symptoms worsen.
☐ Because there were only mild signs and symptoms.
☐ Because I thought it couldn't be COVID19.
☐ Because I was afraid to go to the health service and get contaminated there.
☐ Because he was afraid that his home isolation would be determined.
☐ Because I was afraid to undergo the swab exam
☐ Another motive:________________________________________________________________________________
____________________________________________________________

APPLICATOR, If you checked in item 3.5 HOSPITAL/EMERGENCY ASSISTANCE, read the questions below, if NO, go to 3.8

**3.6** - When looking for the hospital / emergency room, he had to be hospitalized for a day (24 hours) or more?
☐ No (APPLICATOR GO TO 3.8)
☐ Yes, which unit name?____________________________________________
☐ Clinical bed
☐ UTI
☐ Do not know

**3.7** - During this hospital stay, did you need to be sedated, intubated and placed on artificial respiration with a ventilator?
☐ Yes ☐ No ☐ Do not know

**3.8** - How do you evaluate the assistance provided by the health service in which you were assisted?
☐ Good
☐ Regular
☐ Terrible, why?________________________________________________________________________
________________________________________________________________________________________________

**3.9** - How do you evaluate the follow-up of the health professional, to inform you about your test result or the evolution of your signs or symptoms?
☐ There was no follow-up
☐ Good
☐ Regular
☐ Terrible, why? _______________________________________________________________________
________________________________________________________________________________________________

**3.10** - If bad or regular, what can be improved in the care/monitoring of the health service.
☐ Not applicable (if not scored bad or regular)
☐ Describe suggestion:_____________________________________________________________________
________________________________________________________________________________________________

**3.11** - If you had signs or symptoms similar to COVID19 and did NOT seek medical attention, what action did you take to recover from these signs or symptoms? I'll read the options and you say yes, no or you don't know:

☐ Not applicable (he sought medical attention)

| | yes | no | Do not know |
|---|---|---|---|
| Stayed home and did not take medication | | | |
| Stayed home and took self-medication | | | |
| Stayed at home and took homemade medicines | | | |
| Stayed at home and called a health professional or Disk COVID19? | | | |
| Took self-medication and continued with normal activity | | | |
| Did not take medication and continued with normal activity | | | |
| Received a visit from the health professional | | | |
| Any other situation: | | | |

**3.12** - Do you have any medical health plan, whether private, company or public agency?

☐ No ☐ Yes. Which? ___________________________________________________________

**3.13** - Have you done any tests to detect COVID19?

☐ No ☐ Yes, what type of sample was collected?

| **Type of sample**<br>**1- Naso-oropharyngeal swab (nose, throat)**<br>**2- Blood test (with syringe in the arm)**<br>**3- Quick test (finger prick)** | **Collection date** | **Collection location**<br>**Unit/Municipality** | **Result date** | **Test result**<br>**+/-, inconcl., ignored** |
|---|---|---|---|---|
| | | | | |
| | | | | |
| | | | | |
| | | | | |
| | | | | |

**3.14** - Did you take any medication to treat these signs or symptoms that resembled COVID19 BEFORE taking the diagnostic test? APPLICATOR wait for the participant to respond and then help with the options:

☐ No ☐ Yes, which medicine?

☐ Azithromycin ☐ Chloroquine ☐ Ivermectin ☐ Zinc ☐ Vitamin D ☐ Vitamin C

☐ Medicines prescribed at the UPA/Emergency Room/Health Post

☐ Homemade medicines, which? ________________________________

☐ Other, which?

**3.15** - If you've had any symptoms related to COVID19, where do you think you've contracted it? I'll read the options:

☐ At work ☐ Supermarket

☐ At home with family ☐ School/courses

☐ At home with relatives ☐ Church/Cult

☐ Parties / gatherings ☐ Other place__________________________________________________

APPLICATOR, If the participant lives alone (1.11) go to 3.20, otherwise ask the next question

**3.16** – Did people who live or live with you present signs or symptoms related to COVID19 when you became ill?

☐ Yes, BEFORE you experience symptoms ☐ No (APPLICATOR GO TO 3.20)
☐ Yes, AFTER you have symptoms ☐ Don't know (APPLICATOR GO TO 3.20)

**3.17** - Because of this, did they perform any COVID19 tests?

☐ No (APPLICATOR GO TO 3.20)
☐ Yes, which? ______________________________
Test result: ☐ Posit. ☐ Neg. ☐ Don't know
☐ Don't know (APPLICATOR GO TO 3.20)

APPLICATOR, If you marked YES in 3.17, ask the next question, otherwise go to 3.20

**3.18** - Did the people who lived with you comply with social isolation, that is, did they stay at home for a period of 14 days?

☐ Yes ☐ No ☐ Don't know

**3.19** – How often did the person who tested positive receive visits?

☐ Always ☐ Often ☐ Sometimes ☐ Rarely ☐ Never

APPLICATOR, If participant TESTED POSITIVE to COVID19 (3.11), ask next question, if NO go to 3.22.

**3.20** - During your home isolation (indoors) which of these measures did you take?

☐ It was isolated in a room
☐ Clothes were washed separately
☐ Cutlery and glasses were separated from other residents
☐ Used a separate bathroom from other residents, or was sanitized after use
☐ Who took care of you? ______________________________________________________

**3.21** – During home isolation determined by the health service, in which of these situations did you leave home?

☐ Go to the supermarket ☐ Visit relatives
☐ Go to the drugstore ☐ To help someone
☐ Go to the health care service ☐ Go to the country house or farm
☐ To solve work demands ☐ Did not go out during isolation

**3.22** - Did you seek medical attention for any other problem unrelated to the signs and symptoms of COVID19 during the pandemic?

☐ No
☐ Yes, which type?
☐ Routine exams
☐ Obtained medicine
☐ Vaccination
☐ Dentist
☐ Monitor health problem existing before COVID-19.____________________________
☐ Other reason? ____________________________

**3.23** – Which of these other illnesses do you have? I'll read the options and you say yes or no:

| Yes | No | Illness |
|---|---|---|
| | | Hypertension or high blood pressure |
| | | Diabetes or blood sugar |
| | | Asthma or bronchitis |

| Yes | No | Illness |
|---|---|---|
| | | Chronic lung disease |
| | | Immunosuppression |
| | | Câncer |

| | | |
|---|---|---|
| | | Chronic kidney disease |
| | | Chronic heart disease |

| | | |
|---|---|---|
| | | High-risk pregnancy |
| | | Other chronic disease |

**3.24** – Você **descobriu alguma enfermidade** durante a pandemia? Vou ler as opções e você responde sim ou não:

| Yes | No | Illness |
|---|---|---|
| | | Diabetes |
| | | Decompensated diabetes |
| | | Hypertension |
| | | High Cholesterol |
| | | Câncer |

| Yes | No | Illness |
|---|---|---|
| | | Depression |
| | | Anxiety |
| | | Other psychological disorder |
| | | Another unnamed disease? Which one?_______________________________ |
| - | | |
