# Supplementary material for: Prevalence, under-reporting, and epidemiological surveillance of COVID-19 in the Araguaína City of Brazil
Source: PLoS One. 2024 Jun 5;19(6):e0300191. doi: 10.1371/journal.pone.0300191 (PMC11152283; doi:10.1371/journal.pone.0300191)
Supplement: S2 File — (PDF) [file pone.0300191.s002.pdf]

S2 File Materials and methods

Participant selection

The neighborhoods in the urban region of the municipality were grouped into central, northern, southern, eastern, and western regions (Fig 1). The numbers of neighborhoods, blocks, residences, and residents per region were quantified using the geographic recognition (RG2) of the arbovirus control program [1]. Table 1 shows that the sampling units were distributed proportionately to the number of residents in each region.

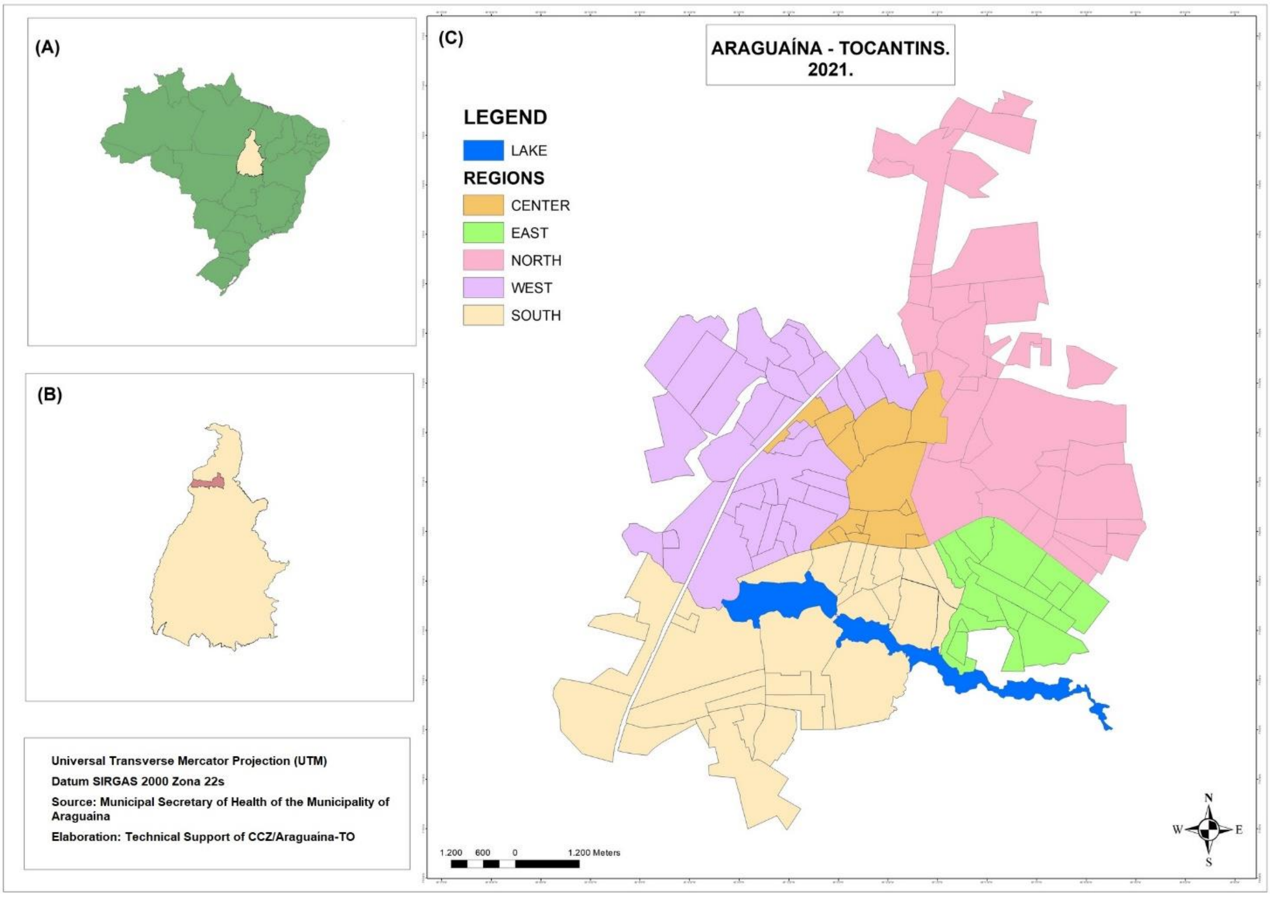

Fig 1. Maps of the Tocantins state. **(A)** Brazil, **(B)** the municipality of Araguaína in Tocantins, and **(C)** Araguaína divided into regions.

Table 1. Sampling and selection of participants according to the residential regions of Araguaína, Tocantins, Brazil, who were asymptomatic for respiratory syndromes.

Region	Estimated population number*	% of population	Minimum number of participants required for the sample	Sample size	% of sample
Center	18,990	10.43	37	80	16.13
North	71,233	39.13	140	181	36.49
South	37,855	20.80	74	105	21.17
East	16,176	8.89	32	49	9.88
West	31,334	17.22	62	82	16.49
Outside the urban area	6,426	3.53	-	-	-
Total	182,014	100%	345	497	100%

*Geographic Recognition (RG2)

Blocks were chosen at random followed by homes. Residents aged ≥18 years were invited to participate. If participation was rejected, the invitation to participate was extended to the next residence. This method was repeated until a predetermined level of acceptance among the residents of these blocks/neighborhoods was achieved. The process of sending out invites to participate in the study was conducted from February 1 to 5, 2021, by endemic agents from the Zoonoses Control Center of the Municipal Secretary of Health from Araguaína (MSHA).

We distributed 608 invitations to residents of selected households, with an estimated non-attendance rate of 18.3%. To ensure the estimated sample size was met, without prejudice to the stratified representativeness, we replaced absent participants with others from the same by region, extending invitations through television media and local websites. Consequently, 497 residents participated in the study, meeting the minimum stipulated sample size for each region, as shown in Table 1.

Data collection

During the sixth epidemiological week of 2021 (February 8 to 12), 497 asymptomatic participants voluntarily visited the study site to complete the epidemiological questionnaire (S2 File Questionnaire). Biological samples were collected during the visit. Participants were verified by their

names, and ages, and residence. The survey data were collected by trained interviewers (undergraduate and graduate students in Animal Health and Public Health in the Tropics from the Federal University of North of Tocantins (UFNT) and in Tropical Medicine and Public Health from the Federal University of Goiás (UFG), while wearing personal protective equipment. The terms of free and informed consent (TFIC) to participate in this study were explained. Only participants who consented and declared themselves asymptomatic (for respiratory syndromes) were included in the study. Participants who showed symptoms suspicious of COVID-19 were instructed to seek medical care in the city and were excluded from the study. After informed consent was provided, the participants were identified using numeric and non-sequential codes to anonymize the data. The survey questionnaire (S2 File) was subdivided into socioeconomic data, preventive measures adopted, and health history. The estimated time for completing of the questionnaire was 14 min.

Serological tests for COVID-19

After completion of the survey, the participants were referred for blood collection for serological testing to quantify IgM and IgG titers. The nursing technician verified the identification document, completed questionnaire, and collected sample following standard procedures marked with the participants' identifier codes. Blood samples with patient identifier codes were sent to a private clinical laboratory, where the serological examination was performed after serum separation in a biosafety environment.

IgM and IgG titers were quantified by chemiluminescence using commercial kits (SARS-CoV-2 IgM Reagent Kit and SARS-CoV-2 IgG Reagent Kit 6R86; Abbott, Sligo, Ireland) registered with the National Health Surveillance Agency. The sensitivity and specificity of the kits were calculated after symptom onset for IgM (sensitivity 88.14%, specificity 99.56%, from 8 to 14 days) and IgG (sensitivity 100%, specificity 99.63%, from ≥14 days), as reported by the manufacturer. The IgG results were considered reactive if the index was ≥1.00 S/CO, indeterminate between 0.8 and

1.00 S/CO, and non-reactive if the index was ≤0.80 S/CO. The IgM results were considered reactive if the index was >1.00 S/CO, and non-reactive if the index was ≤1.00 S/CO. Serological results were attached individually to the survey questionnaire.

RT-qPCR testing for SARS-CoV-2

After blood collection, the participants were directed to the swab collection area. Two nursing professionals provided by the MSHA verified the participants' details and identified the sample transport tubes using the participant codes. Three swabs (rayon tip with plastic stem) were collected from the combined bicavitary areas (oropharyngeal and nasopharyngeal), according to Epidemiological Bulletin No. 01/2020 [1]. The swabs were immediately placed into a 15 mL Falcon tube containing 2 mL sterile saline solution. The samples were refrigerated and were sent twice a day to the Microbiology Laboratory of the School of Veterinary Medicine and Animal Science at UFNT, Araguaína Campus.

In the laboratory, the codes of the participants' samples were crosschecked with those of the signed TFICs. The samples were stored at 7 °C for no more than 24 h before viral ribonucleic acid (RNA) extraction using a commercial kit (QIAamp Viral RNA Mini Kit, Qiagen, Germany) in an NB2 biosafety cabinet. The extracted RNA samples were stored at −20 °C until molecular testing was performed. The extracted RNA products were subjected to RT-qPCR using commercial primers and probes kits from the Centers for Disease Control and Prevention (CDC) (2019NCOV RUO Kit, IDT, USA) targeting two regions of the nucleocapsid phosphoprotein (N) gene and an internal human RNA control in paired uniplex assays on the same plate. A commercial mix (QuantiTect® Probe RT-PCR, Qiagen) with a final volume of 40 µL was used. Amplification reaction was set according to the manufacturer's protocol, and reading in the FAM channel was measured using the QIAquant 96 5plex equipment (Qiagen). Positive controls (2019-nCoV_N and Hs_RPP30 positive controls, IDT) were used for all reactions. The results were interpreted according to the Interim Guidelines for Collecting,

Handling, and Testing Clinical Specimens from Persons for Coronavirus Disease [3]. According to the CDC protocol, a sample was considered positive when the Ct values for N1 and N2 were ≤40. RT-qPCR results were tabulated according to the numeric identifier codes of the participants.

Genome sequencing and phylogenetic analysis

Of the positive RT-qPCR samples, six were selected for genome sequencing. cDNA was synthesized using the Luna Script RT SuperMix (5X) (New England Biolabs, Ipswich, MA, USA). Libraries for whole-virus genome sequencing were prepared according to the ARTIC nCoV-2019 sequencing protocol, version 3 (https://artc.network/ncov-2019). The MinION library was prepared using the Ligation Sequencing kit SQK-LSK-109 and Natives Barcoding kits EXP-NBD104 (Oxford Nanopore, Oxford, UK). The resulting library was loaded onto an R9.4 Oxford MinION flow cell (FLO-MIN106) and sequenced using a MinION Mk1B device. High-accuracy base calling was performed after sequencing the FAST5 files using the Oxford Nanopore Guppy tool (version 3.4.5). The high-accuracy base, called FASTQ files, was assembled using the nCoV-2019 novel coronavirus bioinformatics protocol (https://artic.network/ncov-2019/ncov2019-bioinformatics-sop.html) with minimap2 [4] and medaka (https://github.com/nanoporetech/medaka) for consensus sequence generation.

Pango lineages were attributed to newly assembled genomes using the Pangolin version 3.1.5 software (https://pangolin.cog-uk.io/) [5]. All SARS-CoV-2 Brazilian genomes deposited in GISAID until February 31, 2021, were used to construct a phylogenetic tree. To reduce the genome dataset and allow feasible phylogenetic analysis, a genome sampler was used to select the most closely related samples within the same geographic region and period using our dataset of focal sequences. We then included the reference sequence (NC_045512) to help with the root phylogenies. These steps generated a random sub-sampling of 1,875 SARS-CoV-2 genomes from Brazilian.

The genome sequences were aligned using MAFFT version 7.453 and manually edited using AliView version 1.27 [6]. Maximum-likelihood phylogenies were generated using IQ-TREE version 2.1.2 [7] with the GTR+F+R2 model, as indicated by a substitution model selection analysis conducted with ModelFinder software [8] using 1,000 replicates of ultrafast bootstrapping (−B 1000) and SH-aLRT branch test (−alrt 1000). The ML tree was inspected in TempEst version 1.5.3 [9] to investigate the temporal signal through regression analysis of the root-to-tip genetic distance against sampling dates. A phylogenetic tree was inferred using TreeTime [10]. The time-resolved phylogenetic tree was visualized in R version 4.1.2 using the ggtree package [11].

REFERENCES

1. BRASIL. Diretrizes Nacionais para a Prevenção e Controle de Epidemias de Dengue. Secretaria de Vigilancia em Saúde. Brasília; 2009. Available: http://www.saude.gov.br/bvs
2. BRASIL. Boletim epidemiológico COE nº 01 2020. Ministério da Saúde. Secretaria de Vigilância em Saúde. Brasília, BRASIL: Infecção Humana pelo Novo Coronavírus (2019-nCoV); 2020. p. 17. Available: http://portalarquivos2.saude.gov.br/images/pdf/2020/janeiro/28/Boletim-epidemiologico-SVS-28jan20.pdf
3. CDC. Interim Guidelines for Collecting, Handling and Testing Clinical Specimens from Persons for Coronavirus Disease. 2019. Available: https://www.cdc.gov/coronavirus/2019-ncov/lab/gui delines-clinical-specimens.html
4. Li H. Minimap2: Pairwise alignment for nucleotide sequences. Bioinformatics. 2018;34: 3094–3100. doi:10.1093/bioinformatics/bty191

5. Rambaut A, Holmes EC, O'Toole Á, Hill V, McCrone JT, Ruis C, et al. A dynamic nomenclature proposal for SARS-CoV-2 lineages to assist genomic epidemiology. Nat Microbiol. 2020;5: 1403–1407. doi:10.1038/s41564-020-0770-5

6. Larsson A. AliView: A fast and lightweight alignment viewer and editor for large datasets. Bioinformatics. 2014;30: 3276–3278. doi:10.1093/bioinformatics/btu531

7. Minh BQ, Schmidt HA, Chernomor O, Schrempf D, Woodhams MD, Von Haeseler A, et al. IQ-TREE 2: New Models and Efficient Methods for Phylogenetic Inference in the Genomic Era. Mol Biol Evol. 2020;37: 1530–1534. doi:10.1093/molbev/msaa015

8. Kalyaanamoorthy S, Minh BQ, Wong TKF, Von Haeseler A, Jermiin LS. ModelFinder: Fast model selection for accurate phylogenetic estimates. Nat Methods. 2017;14: 587–589. doi:10.1038/nmeth.4285

9. Rambaut A, Lam TT, Carvalho LM, Pybus OG. Exploring the temporal structure of heterochronous sequences using TempEst (formerly Path-O-Gen). Virus Evol. 2016;2. doi:10.1093/ve/vew007

10. Sagulenko P, Puller V, Neher RA. TreeTime: Maximum-likelihood phylodynamic analysis. Virus Evol. 2018;4. doi:10.1093/ve/vex042

11. Yu G, Smith DK, Zhu H, Guan Y, Lam TTY. ggtree: an r package for visualization and annotation of phylogenetic trees with their covariates and other associated data. Methods Ecol Evol. 2017;8: 28–36. doi:10.1111/2041-210X.12628
